# Advanced optical nanolithography by enhanced transmission through bull’s eye nanostructured meta-mask

**DOI:** 10.1515/nanoph-2023-0145

**Published:** 2023-04-25

**Authors:** Taeyeon Kim, Heesang Ahn, Soojung Kim, Hyerin Song, Jong-ryul Choi, Kyujung Kim

**Affiliations:** Department of Cogno-Mechatronics Engineering, Pusan National University, Busan 46241, Republic of Korea; Medical Device Development Center, Daegu-Gyeongbuk Medical Innovation Foundation (K-MEDI hub), Daegu 41061, Republic of Korea; The Department of Optics and Mechatronics Engineering, Pusan National University, Busan 46241, Republic of Korea; Bio-IT Fusion Technology Research Institute, Pusan National University, Busan 46241, Republic of Korea

**Keywords:** bull’s eye, extraordinary optical transmission, finite-difference time-domain method, nanolithography, optical lithography, plasmonic meta-mask

## Abstract

Plasmonic optical nanolithography using extraordinary optical transmission through a metallic nanohole mask has been actively applied to the high-resolution fabrication of nanostructures over a large area. Although there have been studies on improving the nanostructure fabrication performance in optical nanolithography, such as on adjustable external gap spacing, additional performance enhancement is required for practical applications and commercialization of large-area and high-resolution nanostructure array fabrication techniques. In this study, we design and apply a plasmonic bull’s eye nanostructured meta-mask to enhance the performance of optical nanolithography. Through simulation results and experimental verification, it is confirmed that advanced optical nanolithography using the bull’s eye nanostructured meta-mask has several merits compared to conventional Talbot lithography using nanoholes: (1) Optical nanolithography using the bull’s eye nanostructured meta-mask effectively fabricates nanopillar arrays even at a shorter exposure time than conventional optical lithography using nanoholes. (2) It is possible to create a large-area nanopillar array with various nanopillar diameters by exposure time control in optical nanolithography using the bull’s eye meta-mask. (3) Using water or objective immersion oil to increase the refractive index of the contact medium, light can be focused on smaller sizes, and large-area nanopillar arrays with smaller nanopillar diameters are established. With the upgradation of hardware for large-area fabrication, application of immersion media supplying techniques, and additional studies to establish complex nanostructures, optical nanolithography using the bull’s eye nanostructured meta-mask is an efficient modality to produce various nanostructure-based devices.

## 1 Introduction

With the increase of the utilization of nanophotonic devices in various industrial areas, efficient techniques to fabricate nanophotonic devices over large areas have become important. Photolithography, which is most often employed for nanostructure fabrication, has limitations in the nanostructure size because of the wavelengths of incident light, characteristics of the mask, and performance of the focusing lens that projects patterns onto a sample. Electron beam lithography [1, 2] and focused ion beam lithography [3, 4] are used to fabricate nanostructures with a size smaller than the optical diffraction limits. These methods draw nanostructures one by one, thereby consuming considerable time and cost to fabricate nanophotonic devices over a wide area.

Although various lithographic methods have been improved, optical photolithography is still the most widely used commercial method for fabricating micro-and submicron structures down to a few nanometers. Based on the fundamental physics of the diffraction limits of light, extremely short-wavelength lasers have been employed to fabricate a few nanometers of structures. However, extremely short-wavelength lasers are not only very expensive for broad use, but also the design of optics is difficult for the manipulation of the short-wavelength light path. To overcome the limitations of the low versatility of extreme ultraviolet (EUV) optical photolithography, replaceable lithographic techniques have been actively studied for high-resolution nanostructures in large areas. Among these candidates, plasmonic optical nanolithographic techniques using extraordinary optical transmission (EOT) through metallic nanostructured masks have been actively reported. It can fabricate high-resolution nanostructures without deviation from the conventional photolithographic setup using relatively long-wavelength light excitation [5–9].

For several years, a comparative study of optical lithography based on EOT and conventional photolithography has been conducted to confirm the feasibility of plasmonic lithography for nanoaperture fabrication. We have successfully reported plasmonic lithography for nanostructures below the diffraction limits of light and proposed the possibility of fabricating nanostructures of various shapes with adjustable external gap spacing [8]. Nevertheless, these plasmonic lithographic techniques are still insufficient for commercial use because EOT fields are very sensitive to the optical properties between a metallic mask and excitation light. Thus, EOT fields are too weak to excite photoresistors without perfect plasmon matches between the nanopatterns on the mask and the excitation light wavelength. In most cases, nanohole patterns are used as mask patterns, and extremely highly localized strong EOT fields that define a final fabricated product property cannot be generated at a short wavelength. Thus, it is still necessary to find optimum mask patterns to improve plasmonic fields to excite the photoresist (PR) at the nanoscale.

In this study, we designed a plasmonic meta-mask consisting of multiple bull’s eye nanostructures to overcome these limitations. A bull’s eye nanostructure is a nanoplasmonic optical lens consisting of a central nanoaperture and periodic ring-patterned grooves around the aperture [10–14]. The bull’s eye nanostructure combines incident electromagnetic fields with surface plasmonic polaritons (SPP) propagating along the grooved surface of the lens [11, 15]. It offers an enhancement of the emitted electromagnetic field intensities through bull’s eye meta-lenses and an improvement in the concentration efficiency. Based on this enhancement, the bull’s eye nanostructured meta-mask was designed to generate highly localized and strong excitation fields under diffraction limits. The parameters and processes from this study can be employed for the fabrication of highly resolved nanostructures under diffraction limits using plasmonic lithography with a bull’s eye meta-mask. Additionally, the techniques in this study propose a method of establishing well-designed nanostructures to cover a large area, complementing lithographic systems and the relationship between simulations and practical fabrication results. The results of practical fabrication indicate that nanostructures much smaller than the excitation light wavelength can be fabricated with well-defined plasmonic metalens.

## 2 Materials and methods

### 2.1 Experimental setup for optical nanolithography

**Figure 1:**
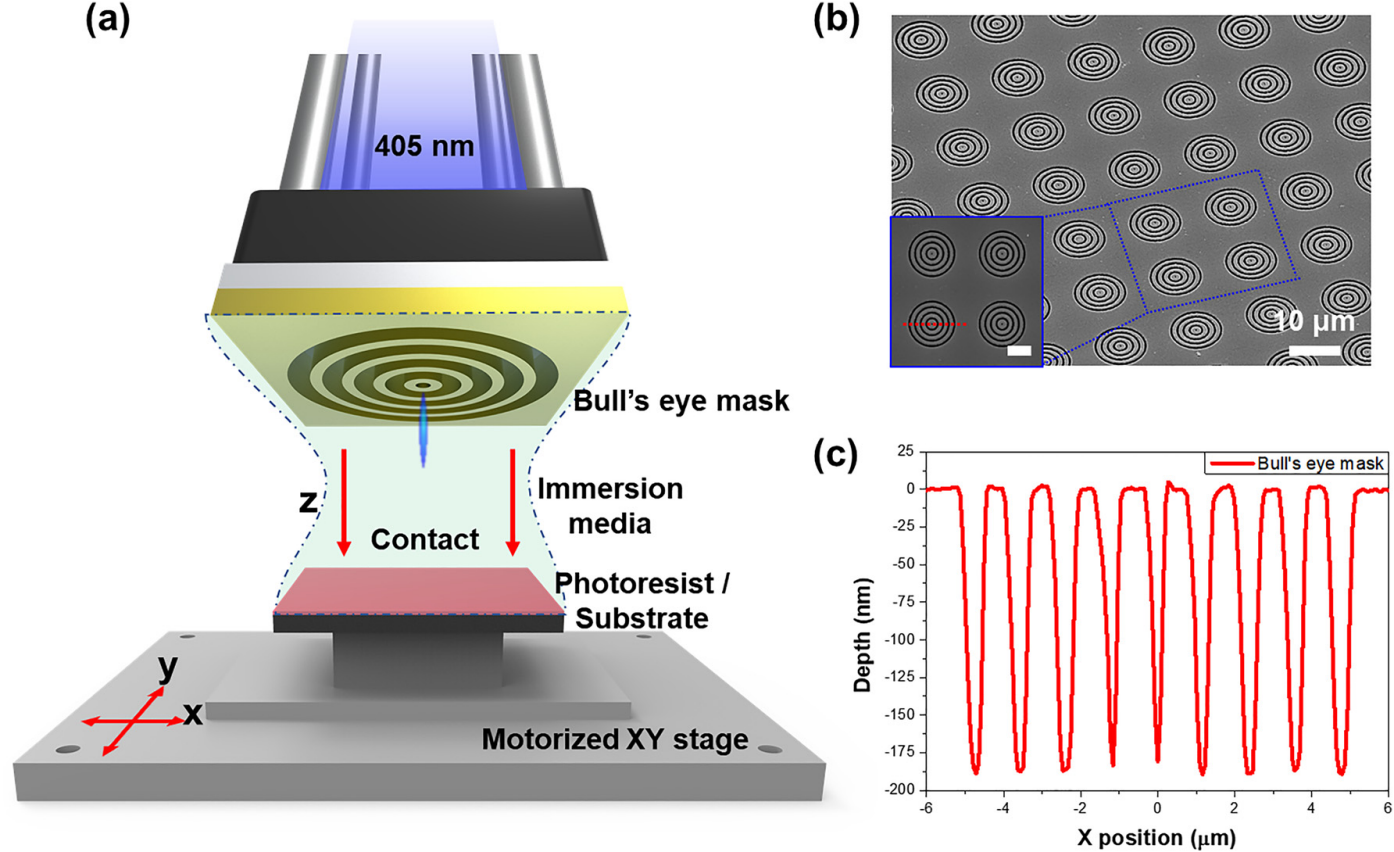
Experimental setup for advanced optical nanolithogrpahy by a bull's eye nanostructured meta-mask. (a) Schematic of an optical nanolithographic system using the bull’s eye nanostructured meta-mask. (b) Tilted scanning electron microscopy (SEM) image of the meta-mask consisting of multiple bull’s eye nanostructures. Each bull’s eye nanostructure consisted of a central nanohole with a diameter of 400 nm and three nanogrooves with a diameter of 600 nm. The period of each nanogroove was also 600 nm. An inlet figure is the SEM image of four bull’s eye nanostructures. A scalebar of the inlet figure indicates 5 μm. (c) Line profile of a depth corresponding to the red line in the inlet figure of Figure 1(b).

The experimental setup for the optical nanolithography is shown in Figure 1(a). The system uses a plasmonic bull’s eye nanostructured lens with an immersion solution. A laser diode (DL5146-101S, Thorlabs, NJ, United States) with a central wavelength of 405 nm served as the light source, and the collimated light was vertically incident on the system through optical components, such as three types of convex lenses, mirrors, an aperture, and a pinhole. The bull’s eye-shaped metalens mask was fixed on the upper stage and positioned to easily contact the positive photoresist (PR) in the *z*-axis direction. The substrate coated with PR was secured using vacuum pump suction from the bottom. Furthermore, a motorized *XY*-axis stage allowed for the free movement of the substrate in the *x*- and *y*-directions, allowing for laser exposure of the desired surface. Prior to contact between the bull’s eye nanostructured lens and PR, the immersion solution was placed between them to evaluate its effect on the refractive index.

### 2.2 Theory of bull’s eye shaped metalens and simulation of enhanced electromagnetic fields through the bull’s eye

Generally, the electromagnetic fields and properties of SPP that occur at a metal-dielectric interface can be estimated using Maxell’s equations [15–17]. SPP is a transverse magnetic wave, and an SPP wave that satisfies Maxwell’s equations also satisfies the following equations:

$$\frac{k_{zm}}{\varepsilon_{m}}+\frac{k_{zd}}{\varepsilon_{d}}=0$$

$$k_{x}^{2}+k_{zm}^{2}=\varepsilon_{m}{\left(\frac{\omega }{c}\right)}^{2}$$

$$k_{x}^{2}+k_{zd}^{2}=\varepsilon_{d}{\left(\frac{\omega }{c}\right)}^{2}$$

*k*_*x*_ and *k*_*z*_ are the *x*- and *z*-components of the wave vector (*k*), respectively. *k*_*x*_ is the same in both the metal and dielectric, but *k*_*z*_ of the metal and *k*_*z*_ of the dialectic are different. Therefore, *k*_*z*_ of the metal is expressed as *k*_*zm*_, and *k*_*z*_ of the dielectric is expressed as *k*_*zd*_. *ɛ*_*m*_ and *ɛ*_*d*_ indicate the permittivities of the metal and the dielectric, respectively. *ω* and *c* are the angular frequency and speed of the light, respectively. From the above equations, the wave vector of the SPP that propagates on the surface of the metal-dielectric interface is calculated as follows:

$$k_{x}=\frac{\omega }{c}\sqrt{\frac{\varepsilon_{m}\varepsilon_{d}}{\varepsilon_{m}+\varepsilon_{d}}}$$

Additionally, the coupling condition of electromagnetic waves incident orthogonally to the substrate by grooved grating patterns can be briefly expressed as follows [18]:

$$k_{x}=m\frac{2\pi }{\Lambda }$$

Λ and *m* indicate a period of grooved grating patterns and diffraction order.

We applied a finite-difference time-domain (FDTD) method to calculate the enhanced electromagnetic fields through the bull’s eye-shaped metalens based on the theoretical equations above. FDTD solves Maxwell’s equations in two- or three-dimensions, which allows the calculation of **E** (electrical fields) and **H** (magnetic fields) for each unit area. FDTD can estimate **E** and **H** in each unit area using the permittivity, permeability, and wavenumber. The light incident on the nanophotonic device was calculated using the FDTD method. In this study, we utilized commercial FDTD software (FDTD Solution, Lumerical, Inc., PA, United States) for the simulations. The fixed geometric parameters in this calculation included a pitch of 15 µm × 15 µm, a total gold thickness of 200 nm, and hole and groove depths of 190 nm for both the nanohole arrays and bull’s eye structure. Furthermore, an adaptive mesh and perfectly matched layer (PML) boundary condition were employed in the out-of-plane direction along the *z*-axis. Symmetric and antisymmetric boundary conditions were selected in the *x*- and *y*-directions for computational simplicity. Each structure was illuminated by a plane wave with a central wavelength of 405 nm.

### 2.3 Fabrication of metallic bull’s eye nanostructure masks

The bull’s eye nanostructured meta-mask and EOT mask were fabricated on a BK7 substrate glass using an electron beam and thermal deposition using a vacuum evaporator (SHE-6T-350D, SAMHAN VACUUM TECH. Co., Ltd., Seoul, Republic of Korea). The process involved depositing 5 nm of chrome (Cr) followed by 10 nm of gold (Au), at a deposition rate of 0.7 Å/s and 1.0 Å/s, respectively, in a 10^−6^ mbar chamber pressure. Cr and Au were used as the adhesive and conductive layers, respectively. For the lithography process, the substrate was spin-coated with a negative electron beam resist (Ma-N-2403, MicroChem, MA, United States) at a spin rate of 500 rpm for 5 s and 4000 rpm for 45 s, followed by hot-plate baking at 100 °C for 2 min. The thickness of the electron beam resist was 200 nm. The bull’s eye-shaped metalens and nanohole array patterns were formed using a 30 keV electron beam lithography system (Draw beam system, VEGA3, TESCAN, Brno, Czech Republic). After development using an aqueous-alkaline-based developer for resists (ma-D 525, MicroChem, MA, United States) and cleaning, a 190 nm Au layer was deposited by thermal evaporation at a rate of 1.0 Å/s and at a 10^−6^ mbar pressure. The lift-off process was performed using acetone for 30 min on a sonication machine to remove any residue and reveal the bull’s eye and EOT structure. Finally, the bull’s eye nanostructured meta-masks and EOT mask were cleaned by a washing process in ethyl alcohol and deionized water for use in the experiment.

### 2.4 Optical lithography processes

A 20 mm × 20 mm silicon wafer was used as the substrate in all experiments for stability. A positive photoresist (AZ GXR-601, AZ Electronic Materials, Luxembourg, Germany), sensitive to 405 nm light, was spin-coated onto the cleaned substrate at a spin rate of 500 rpm for 5 s and 8000 rpm for 45 s. Subsequently, it was baked at 115 °C for 3 min to evaporate the solvent. The photoresist was then subjected to different immersion media, resulting in varying electric-field distributions for an immersion objective lens. In the air media condition experiment, the substrate and mask were in direct contact. For the immersion media condition experiment, 5 μL of the immersion solution was pipetted onto the photoresist-coated substrate before contact with the substrate and mask. The exposure time was precisely controlled at 3–15 s (for the bull’s eye nanostructured meta-mask) and 5–15 min (for the EOT mask) using the external modulation mode function of the programmable shutter. The light-exposed areas of the samples were then removed using a photoresist developer (AZ 300 MIF, AZ Electronic Materials, Luxembourg, Germany) and deionized water for 1 min each. A 50 nm Au layer was deposited using evaporation equipment, and any unwanted areas were removed using a lift-off process to obtain a nanopillar structure. Finally, the shapes and sizes of the fabricated nanopillars were analyzed and compared using scanning electron microscopy (SEM, VEGA3, TESCAN, Brno, Czech Republic).

## 3 Results

### 3.1 Electromagnetic fields stimulation of optical nanolithography using bull’s eye nanostructured masks

Prior to experimental optical nanolithography using nanostructured masks, electromagnetic field distributions through a nanohole and a bull’s eye nanostructured meta-mask were theoretically calculated. To establish optimized bull’s eye nanostructured meta-mask to generate concentrated electromagnetic fields with a high efficiency, the difference in electromagnetic fields distributions according to a number of ring apertures constituting the bull’s eye meta-mask and a width of each ring aperture were compared as Figures S1 and S2. As described in the result of Figure S1, it was confirmed that at least four ring apertures should be in the bull’s eye nanostructured meta-mask to perform nanolithography by concentrated optical transmission. According to the result in Figure S2, the width of each ring aperture does not significantly affect the distance at which optical transmission is focused, but an intensity of the concentrated optical transmission is the highest when the width of each ring aperture is 400 nm. Based on these simulation results, an optimized bull’s eye nanostructured meta-mask was designed and used in electromagnetic fields distributions calculations according to changes in refractive indices of superstrate.

**Figure 2:**
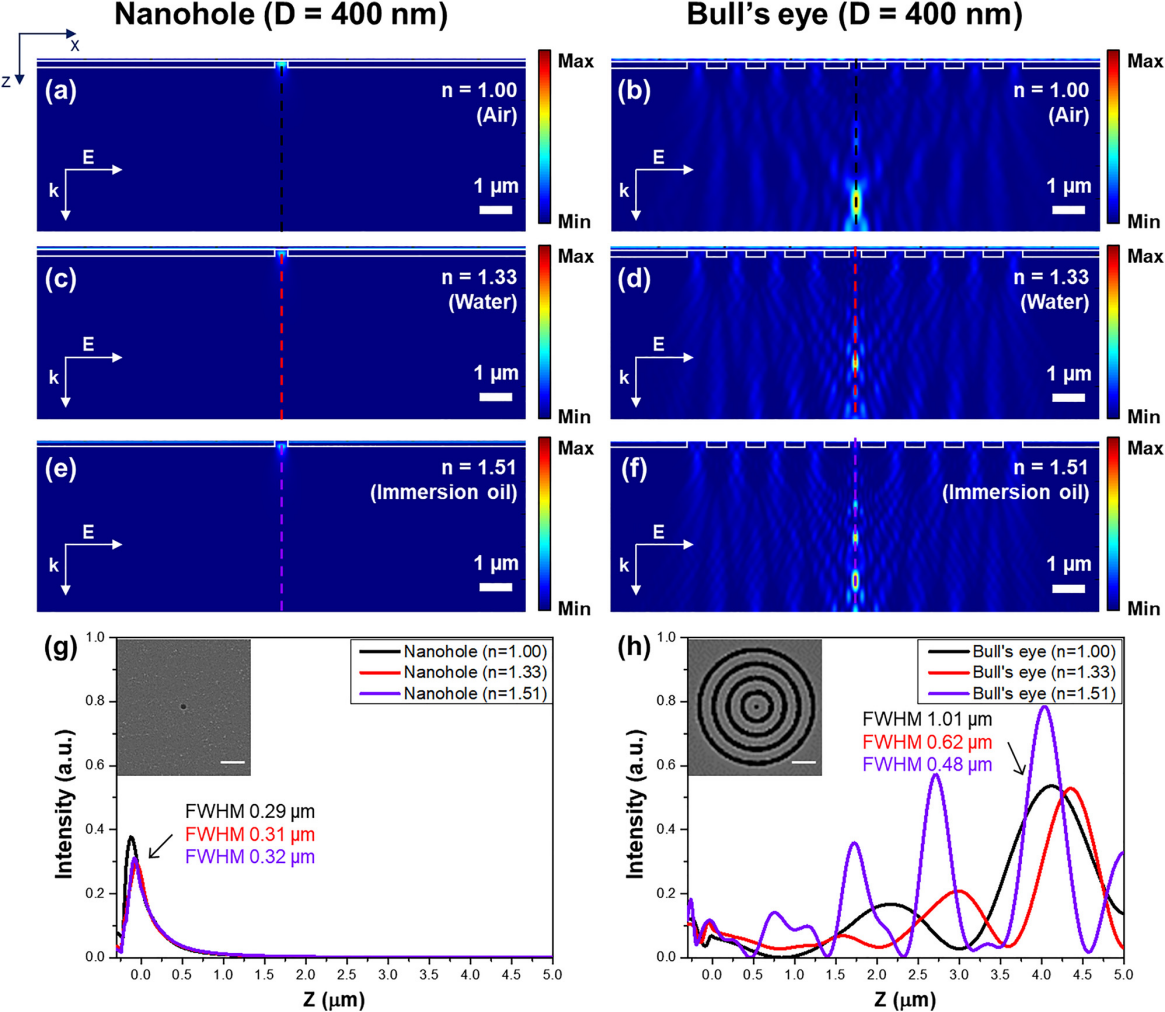
Simulation results of electromagnetic fields distributions of optical transmission through a nanohole and a bull's eye nanostructured meta-mask. (a and b) Electromagnetic fields distributions of optical transmission through (a) the nanohole with a diameter of 400 nm and (b) bull’s eye meta-mask in a condition of air as a contact medium. (c and d) Electromagnetic fields distributions of optical transmission through (c) the nanohole and (d) bull’s eye meta-mask with water immersion. (e and f) Electromagnetic fields distributions of optical transmission through (e) the nanohole and (f) bull’s eye meta-mask with an objective immersion oil as a contact medium. (g and h) Intensity profiles of optical transmission through (e) the nanohole and (f) bull’s eye nanostructured meta-mask. Scale bars in inlet figures of Figure 2(g) and (h) indicate 2 μm.

Figure 2(a–f) show the electromagnetic field distributions of optical transmission through a nanohole with a diameter of 400 nm and a bull’s eye nanostructured meta-mask with a central hole diameter of 400 nm. The period and size of the nanogrooves around the central hole in the bull’s eye nanostructure were 600 nm. Figure 2(a and b) show the simulation results when the superstrate is air (*n* = 1.00), and Figure 2(c and d) show the results when the superstrate is water (*n* = 1.33) with a higher refractive index than air. Also, Figure 2(e and f) indicate the calculated electromagnetic fields distributions when the superstrate is objective immersion oil (*n* = 1.51). It can be observed from the simulation results in Figure 2(a), (c), and (e) that the optical transmission by the nanohole has the highest intensity near the surface of the substrate where the nanohole was fabricated. This implies that an efficient fabrication process is possible only when the nanohole-integrated mask and the sample to be integrated with the nanopillar array are in close contact. On the other hand, as described in Figure 2(b), (d), and (f), light is reinforced through the central nanohole and nanogrooves in the bull’s eye nanostructured meta-mask to form a focused beam spot. This beam spot is formed at a distance of 4.0–4.5 μm from the surface of the bull’s eye nanostructure, which is a distance that can be sufficiently controlled using conventional optical micro-stages. Compared to optical transmission through the nanohole reaching the same distance, the focused beam spot through the bull’s eye nanostructured meta-mask is enhanced by approximately 902 times in a condition of air as a contact medium and 646 times in water immersion. In case of objective immersion oil as a contact media, an enhancement factor of the focused beam spot through the bull’s eye nanostructured meta-mask is approximately 951 times. This means that nanopillar array fabrication is possible even if it requires considerably less exposure time than nanohole-based Talbot lithography at a certain distance. It can also be confirmed in Figure S3, a simulation result considering layers of a photoresist and a substrate. As described in the result in Figure S3, a highly concentrated optical transmission is delivered to the photoresist layer when the photoresist layer is positioned at a height at which electromagnetic fields is focused by the bull’s eye nanostructured meta-mask. Moreover, when an optical photoresist causes a chemical reaction by irradiation with more than a certain amount of optical energy, the diameter of the fabricated nanopillars can be adjusted by adjusting the exposure time.

Figure 2(e) and (f) describe the central line profiles of each electromagnetic field distribution of the optical transmission. As shown by the line profiles and full width half maximum of concentrated electromagnetic fields in Figure 2(f), optical transmission can be tightly focused to a smaller size when the refractive index of the superstrate (contact media) is increased by water and objective immersion oil immersions. This indicates that optical nanolithography using superstrate media with higher refractive index than air can be used to fabricate nanopillars with a smaller diameter.

### 3.2 Experimental results of advanced nanolithography by enhanced optical transmission through bull’s eye nanostructured masks

Through the simulation results and analysis, we derived the characteristics and merits of optical nanolithography using the bull’s eye nanostructured meta-mask as follows: (1) optical nanolithography using the bull’s eye nanostructured meta-mask can effectively fabricate nanopillar arrays even at a shorter exposure time than conventional optical lithography using nanoholes. (2) It is possible to create a large-area nanopillar array with various nanopillar diameters by exposure time control in optical nanolithography using the bull’s eye meta-mask. (3) Using water immersion to increase the refractive index of the contact medium, light can be focused on smaller sizes, and large-area nanopillar arrays with smaller nanopillar diameters can be established. The advantages and features of optical nanolithography using the bull’s eye meta-mask were experimentally confirmed by configuring the optical nanolithographic system (as shown in Figure 1(a)) and applying the bull’s eye nanostructured meta-mask (as described in Figure 1(b)).

**Figure 3:**
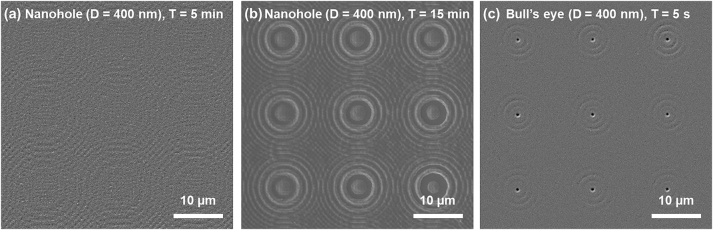
Scanning electron microscopy (SEM) images of developed photoresist by optical transmission through an array of nanoholes and a bull's eye nanostructured meta-mask. (a and b) SEM images of developed photoresist by optical transmission through an array of nanoholes with exposure times of (a) 5 min and (b) 15 min. Chemical reactions and developments of photoresists were not observed in optical lithography by the nanoholes. In the case of 15 min of the exposure time, the development of photoresist to create nanopillars did not occur, and only an airy pattern was remarkable. (c) SEM image of photoresist developed by optical transmission through a bull’s eye nanostructured meta-mask with an exposure time of 5 s. Unlike the conventional optical lithography using the nanoholes, the development of photoresists for the nanopillars array fabricated was well-achieved with reduced exposure time.

Figure 3 shows the result (SEM images) of comparing the photoresists developed by optical transmission through the nanoholes and bull’s eye nanostructured meta-mask. As described in Figure 3(a), the chemical reactions and development of photoresists were not observed in optical lithography by the nanoholes with an exposure time of 5 min. Even in the case where the exposure time of 15 min was given (Figure 3(b)), the development of a photoresist to create nanopillars did not occur, and only an airy pattern was remarkable. However, the development of photoresists for nanopillar array fabrication was confirmed in advanced optical nanolithography using the bull’s eye nanostructured meta-mask with an exposure time of 5 s (Figure 3(c)). The results imply that an optical nanolithographic system capable of fabricating large-area nanostructure arrays at a higher fabrication speed is possible using a bull’s eye nanostructured meta-mask.

**Figure 4:**
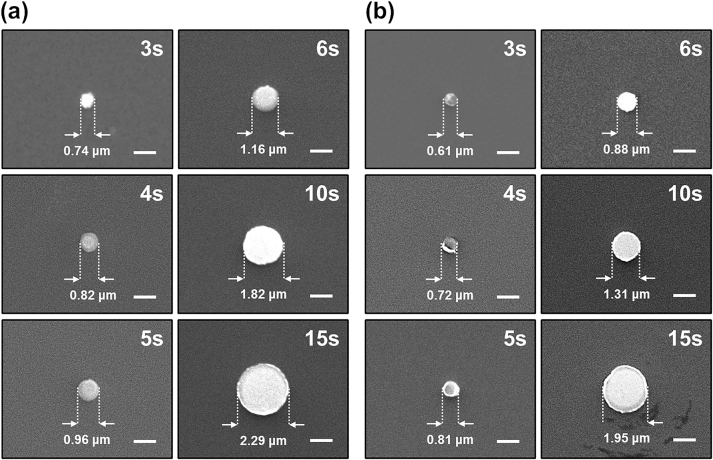
Comparative result of air and water as a contact medium in optical nanolithography using a bull’s eye nanostructured meta-mask. (a and b) Scanning electron microscopy (SEM) images of nanopillars fabricated by optical nanolithography using the bull’s eye nanostructured meta-mask (a) in a condition of air as a contact medium and (b) with water immersion.

**Figure 5:**
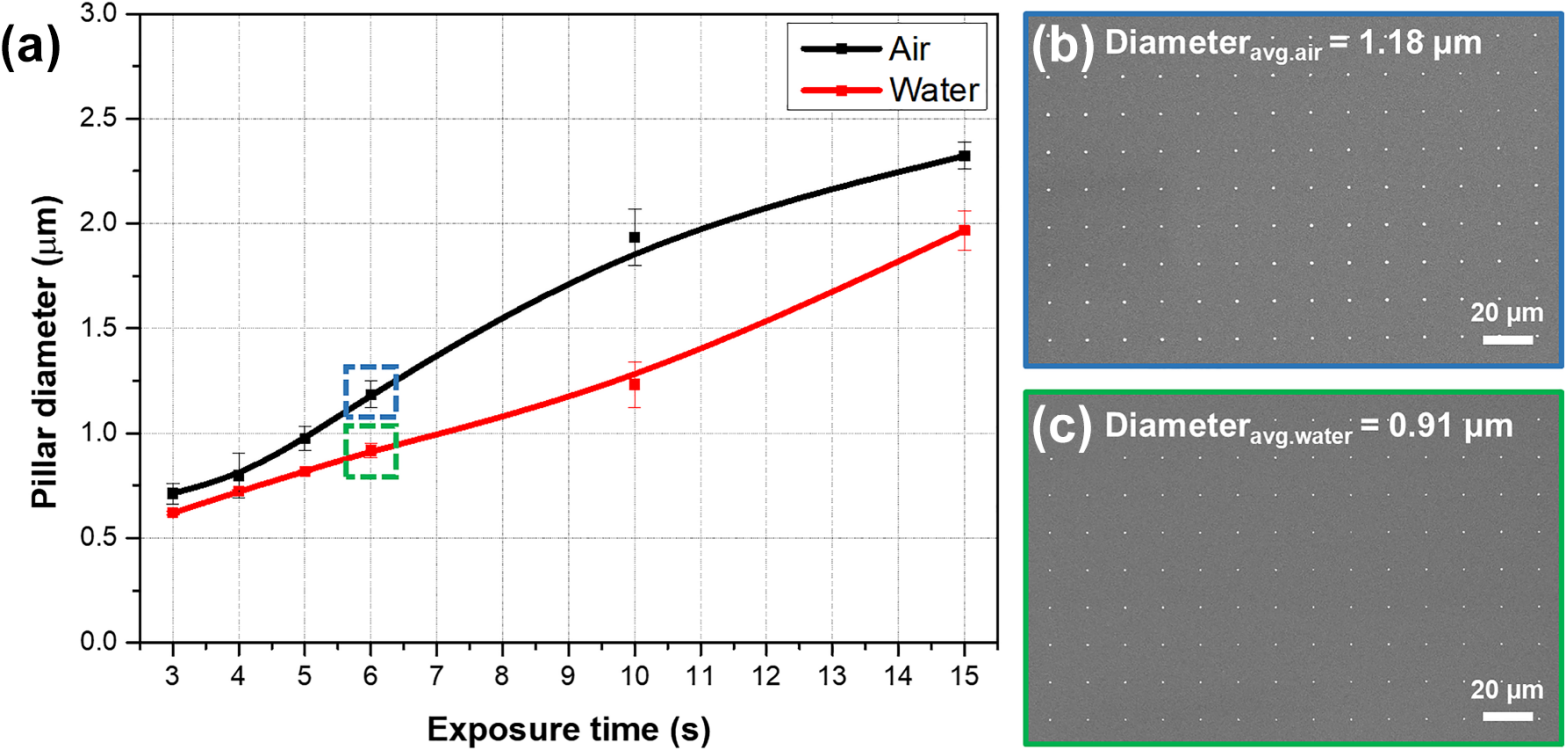
Comparision of fabricated nanopillars by optical nanolithogrpahy using a bull's eye nanostructured meta-mask in coditions of air and water as a contact medium. (a) Quantitative analysis of the relationship of fabricated nanopillar diameters (μm) via exposure times (s) using optical nanolithography based on a bull’s eye nanostructured meta-mask. (b and c) Scanning electron microscopy (SEM) images of a large-area nanopillars array established by optical nanolithography using a meta-mask in which multiple Bull’s eye nanostructures were integrated into an array. The contact medium and exposure time in Figure 5(b) were air and 6 s. In Figure 5(c), water immersion and 6 s as the exposure time were applied.

Subsequently, nanopillar arrays were fabricated by optical nanolithography using a bull’s eye nanostructured meta-mask with different exposure times. Figure 4 shows the SEM images of nanopillars fabricated by bull’s eye meta-mask-based advanced optical nanolithography with different exposure times (3–15 s) under conditions of air and water immersion. In a condition of air immersion as a contact medium (Figure 4(a)), the diameters of the fabricated nanopillars with exposure times of 3, 4, 5, 6, 10, and 15 s were 0.74, 0.82, 0.96, 1.16, 1.82, 2.29 μm, respectively. Under water immersion (Figure 4(b)), the nanopillars fabricated with exposure times of 3, 4, 5, 6, 10, and 15 s were 0.61, 0.72, 0.81, 0.88, 1.31, 1.95 μm, respectively. As expected from the simulation results, differences in the diameters of the fabricated nanopillars occurred owing to changes in the exposure time of the optical nanolithography, and the diameter increased as the exposure time increased. Moreover, it was experimentally confirmed that increasing the refractive index in the contact medium using water immersion enables the production of nanopillars with smaller diameters. When comparing the diameters of nanopillars fabricated by bull’s eye meta-mask-based optical nanolithography under water immersion versus air immersion at each exposure time, offering a higher refractive index using water immersion enables the fabrication of nanopillars, which are on average 19.3 % smaller. This can be confirmed in more detail in Figure 5(a), which describes the relationship of the fabricated nanopillar diameters (μm) via exposure times (seconds) using advanced optical nanolithography based on the bull’s eye nanostructured meta-mask.

Additionally, a large-area nanopillar array was established by optical nanolithography with a meta-mask in which multiple bull’s eye nanostructures were integrated into an array. Figure 5(b) and (c) are SEM images of the nanopillar arrays that were fabricated in a large area using arrayed multiple bull’s eye nanostructures under air and water immersion as a contact medium, respectively. The exposure time for fabricating the nanopillars in Figure 5(b) and (c) was 6 s. Through these SEM images and analyses, it was confirmed that uniform and large-area nanopillar arrays can be fabricated by advanced optical nanolithography using arrayed meta-masks of a bull’s eye nanostructure.

### 3.3 Discussion

As confirmed in the findings of the previous section, advanced optical nanolithography using the bull’s eye nanostructured meta-mask is a technique that can effectively fabricate large-area nanopillar arrays with a dramatically reduced exposure time. An optical nanolithographic system based on the bull’s eye meta-mask is advantageous for the effective fabrication of various large-area photonic devices that consist of nanostructures.

Talbot lithography, based on Talbot interference [19], has merits of being able to fabricate microscale and sub-microscale structures over a large area and simplifying nanostructure lithographic system. Since a Talbot image, which is regularly repeated for each Talbot length, is formed by a grating component in Talbot lithography, a photoresist-coated sample should be located at a height with an interference pattern corresponding to the desired structure. Too short working distance between the grating and the sample can distort the fabricated pattern due to interference, and too long working distance has a disadvantage of being too week to effectively fabricate structures [20, 21]. In this study, a lithographic system using concentrated plasmonic fields at a certain height using the Bull’s eye meta mask to fabricate a nanopillars array in a large area. Like Talbot lithography, the working distance between the meta-mask and the sample is important in plasmonic lithography. When the working distance is not precisely controlled and matched to the height for the concentrated plasmonic fields, the efficiency of nanopillars fabrication would be greatly reduced. It shows the need for precise adjustments of the working distance in both Talbot lithography and plasmonic lithography using the Bull’s eye meta-mask.

To increase the efficiency of the nanostructure and large-area photonic device fabrication, it is necessary to apply a method of simultaneous light exposure to a larger area using a wider bull’s eye nanostructured meta-mask. It requires the fabrication of a wider bull’s eye nanostructure array and collimated light illumination techniques capable of evenly incident light into a large area. Additionally, the optical nanolithographic system can fabricate nanopillars in a large area while moving the meta-mask and light illumination modules by high-speed motorized stages. The integration of optical inspection modules for the simultaneous fabrication and inspection of nanostructures is a helpful technique for evaluating an advanced optical nanolithographic system.

In this study, it was confirmed that water and objective immersion oil allow the fabrication of smaller nanopillars by increasing the refractive index between the meta-mask and substrate on which the nanopillars were established. According to the results, solutions with a higher refractive index than water can be applied to fabricate photonic devices composed of smaller nanopillars. To supply a solution with a high refractive index as a contact medium, additional devices such as fluidic supply modules should be included in the optical nanolithographic system [22, 23]. Additionally, the development of devices and techniques that can effectively remove the solution to provide a high refractive index in the contact medium after fabrication should be applied during the post-processing stage.

As a representative nanostructure, a large-area nanopillar array was fabricated using a bull’s eye nanostructured meta-mask and an it-integrated optical nanolithographic system. In addition to the nanopillar arrays, the design of various meta-masks, calculation of electromagnetic field distributions of other meta-masks, and experimental verification are required to fabricate various complex nanostructures [24–29], which have been used in practical photonic components and metamaterials. Modalities to control the shapes of nanostructures developed by enhanced optical transmission using fine movements of the bull’s eye nanostructured meta-mask or structured light need to be studied for the development of advanced optical nanolithographic systems using nanostructured meta-masks.

## 4 Concluding remarks

We studied the feasibility of optical lithography based on EOT and diffracted light transmission with an external gap spacing. Both EOT with nanoapertures smaller than the wavelength of the incident light and diffracted transmission through nanoholes larger than the wavelength can be employed for fabricating nanostructures by optical lithography. By comparing the calculated electromagnetic field distributions and fabricated patterns of the nanostructures, we confirmed that the simulation results could be applied to a design for nanostructure fabrication. Additionally, it was determined that the light transmitted from the nanohole array had different shapes depending on the height from the mask surface. Therefore, nanostructure fabrication in optical lithography needs to be adjusted for the exact height. In terms of enhancing the usability, height adjustments by external gap spacing can be used to create nanostructures of various shapes by optical lithography. Although there are several issues to be addressed, such as the optimization of the exposure time and analysis of electromagnetic field distributions from various nanostructures of the mask, the technologies evaluated in this study propose a way to establish well-designed nanostructures over a large area.

## Supplementary Material

Supplementary Material Details
