# Supplementary material for: Advanced optical nanolithography by enhanced transmission through bull’s eye nanostructured meta-mask
Source: Nanophotonics. 2023 Apr 25;12(11):2041–50. doi: 10.1515/nanoph-2023-0145 (PMC11501246; doi:10.1515/nanoph-2023-0145)
Supplement: Supplementary file 1 — Supplementary Material Details [file j_nanoph-2023-0145_suppl.docx]

**Taeyeon Kim^1^, Heesang Ahn^1^, Soojung Kim^1^, Hyerin Song^1^, Jong-ryul Choi^2,^*, Kyujung Kim^1,3,*^**

^1^Department of Cogno-Mechatronics Engineering, Pusan National University,
Busan 46241, Republic of Korea

^2^Medical Device Development Center, Daegu-Gyeongbuk Medical Innovation Foundation (K-MEDI hub), Daegu 41061, Republic of Korea

^3^The Department of Optics and Mechatronics Engineering, Pusan National University,
Busan 46241, Republic of Korea

^*^Corresponding author:
K. Kim ([k.kim@pusan.ac.kr](mailto:k.kim@pusan.ac.kr)); J. Choi ([jongryul32@kmedihub.re.kr](mailto:jongryul32@kmedihub.re.kr))

Advanced optical nanolithography by enhanced transmission through bull’s eye nanostructured meta-mask

**Supplementary Information**

Abstract: Plasmonic optical nanolithography using extraordinary optical transmission through a metallic nanohole mask has been actively applied to the high-resolution fabrication of nanostructures over a large area. Although there have been studies on improving the nanostructure fabrication performance in optical nanolithography, such as on adjustable external gap spacing, additional performance enhancement is required for practical applications and commercialization of large-area and high-resolution nanostructure array fabrication techniques. In this study, we design and apply a plasmonic bull’s eye nanostructured meta-mask to enhance the performance of optical nanolithography. Through simulation results and experimental verification, it is confirmed that advanced optical nanolithography using the bull’s eye nanostructured meta-mask has several merits compared to conventional Talbot lithography using nanoholes: (1) Optical nanolithography using the bull’s eye nanostructured meta-mask effectively fabricates nanopillar arrays even at a shorter exposure time than conventional optical lithography using nanoholes. (2) It is possible to create a large-area nanopillar array with various nanopillar diameters by exposure time control in optical nanolithography using the bull’s eye meta-mask. (3) Using water or objective immersion oil to increase the refractive index of the contact medium, light can be focused on smaller sizes, and large-area nanopillar arrays with smaller nanopillar diameters are established. With the upgradation of hardware for large-area fabrication, application of immersion media supplying techniques, and additional studies to establish complex nanostructures, optical nanolithography using the bull’s eye nanostructured meta-mask is an efficient modality to produce various nanostructure-based devices.

Keywords: Optical lithography, Nanolithography, Bull’s eye, Plasmonic meta-mask, Extraordinary optical transmission, Finite-difference time-domain method


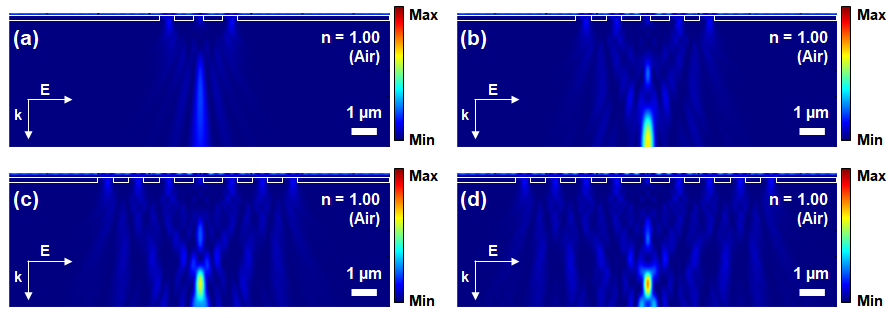


Fig. S1: Electromagnetic fields distributions of optical transmission through a bull’s eye nanostructured meta-mask with (a) a single ring aperture, (b) two ring apertures, (c) three ring apertures, and (d) four ring apertures in a condition of air as a contact medium. At least four ring apertures should be in the bull’s eye nanostructured meta-mask to generate concentrated optical transmission.


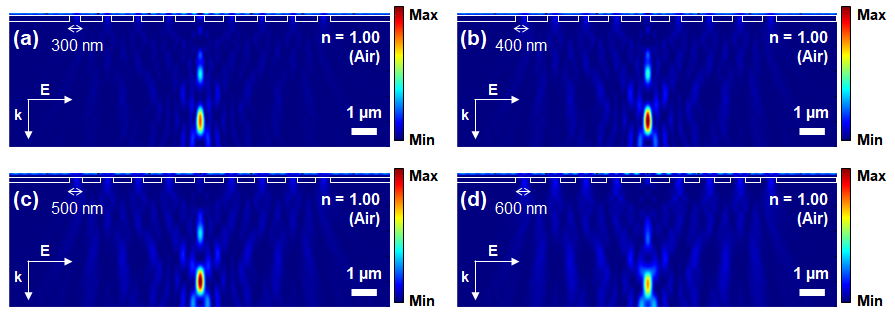


Fig. S2: Electromagnetic fields distributions of optical transmission through a bull’s eye nanostructured meta-mask with different widths of ring apertures in a condition of air as a contact medium. (a) 300 nm, (b) 400 nm, (c) 500 nm, and (d) 600 nm widths of each ring aperture.


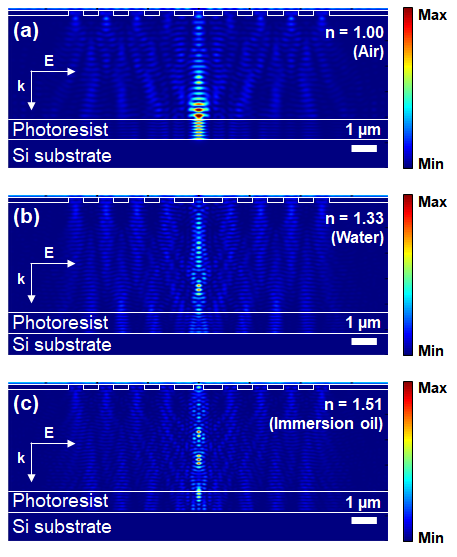


Fig. S3: Simulation results of electromagnetic fields distributions of optical transmission through a bull’s eye nanostructured meta-mask with a consideration of layers of a photoresist and a substrate. A superstrate between the bull’s eye meta-lens and the photoresist were (a) air, (b) water, and (c) an objective immersion oil.
